# Estimating the Burden of Malaria in Senegal: Bayesian Zero-Inflated Binomial Geostatistical Modeling of the MIS 2008 Data

**DOI:** 10.1371/journal.pone.0032625

**Published:** 2012-03-05

**Authors:** Federica Giardina, Laura Gosoniu, Lassana Konate, Mame Birame Diouf, Robert Perry, Oumar Gaye, Ousmane Faye, Penelope Vounatsou

**Affiliations:** 1 Department of Epidemiology and Public Health, Swiss Tropical and Public Health Institute, Basel, Switzerland; 2 University of Basel, Basel, Switzerland; 3 Faculté des Sciences et Techniques, UCAD Dakar, Sénégal; 4 National Malaria Control Programme, Dakar, Sénégal; 5 Center for Global Health, Centers for Disease Control and Prevention, Atlanta, Georgia, United States of America; 6 Faculté de Médecine, Pharmacie et Odontologie, UCAD Dakar, Sénégal; London School of Hygiene and Tropical Medicine, United Kingdom

## Abstract

The Research Center for Human Development in Dakar (CRDH) with the technical assistance of ICF Macro and the National Malaria Control Programme (NMCP) conducted in 2008/2009 the Senegal Malaria Indicator Survey (SMIS), the first nationally representative household survey collecting parasitological data and malaria-related indicators. In this paper, we present spatially explicit parasitaemia risk estimates and number of infected children below 5 years. Geostatistical Zero-Inflated Binomial models (ZIB) were developed to take into account the large number of zero-prevalence survey locations (70%) in the data. Bayesian variable selection methods were incorporated within a geostatistical framework in order to choose the best set of environmental and climatic covariates associated with the parasitaemia risk. Model validation confirmed that the ZIB model had a better predictive ability than the standard Binomial analogue. Markov chain Monte Carlo (MCMC) methods were used for inference. Several insecticide treated nets (ITN) coverage indicators were calculated to assess the effectiveness of interventions. After adjusting for climatic and socio-economic factors, the presence of at least one ITN per every two household members and living in urban areas reduced the odds of parasitaemia by 86% and 81% respectively. Posterior estimates of the ORs related to the wealth index show a decreasing trend with the quintiles. Infection odds appear to be increasing with age. The population-adjusted prevalence ranges from 0.12% in Thillé-Boubacar to 13.1% in Dabo. Tambacounda has the highest population-adjusted predicted prevalence (8.08%) whereas the region with the highest estimated number of infected children under the age of 5 years is Kolda (13940). The contemporary map and estimates of malaria burden identify the priority areas for future control interventions and provide baseline information for monitoring and evaluation. Zero-Inflated formulations are more appropriate in modeling sparse geostatistical survey data, expected to arise more frequently as malaria research is focused on elimination.

## Introduction

More than two hundred million cases of malaria were estimated worldwide in 2008 and the majority (85%) was in African countries. Malaria accounted for 850 thousand deaths in the same year, 89% of which occurred in Africa. Over 85% of deaths were in children under five years of age [1]. Senegal is one of the 45 countries in Africa where malaria is endemic and represents the leading cause of morbidity and hospital mortality [2]. The main parasite transmitted by anopheline mosquitoes is *Plasmodium falciparum* and transmission occurs seasonally in the entire country, from June to November. Rapid diagnostic tests (RDTs) have been provided free of charge since 2007. Two years later, almost 86% of suspected malarial fever cases were screened with an RDT [3]. Malaria incidence in children under five decreased from 400 000 suspected cases in 2006 to 30 000 confirmed cases in 2009 [4]. Routine surveillance provides some evidence that the number of malaria inpatient cases and deaths during the same period are decreasing. However, these estimates must be interpreted with caution since they are affected by poor reporting, introduction of RDTs as well as changes in case definition [1]. Furthermore, the lack of nationally representative surveys makes these estimates unreliable. Almost all malaria surveys in Senegal were carried out in five parts of the country: Dakar and its suburbs, specific areas around the Senegal River, Fatick region and Niakhar province. Few studies have been conducted in the rest of the country, particularly in the regions of Tambacounda and Casamance.

The Senegal Malaria Indicator Survey (SMIS) is the second nationally representative household survey focusing on malaria-related indicators and the first that collected parasitological data. The survey was supported by the National Malaria Control Program (NMCP) and carried out between November 2008 and January 2009 by the Research Center for Human Development in Dakar (CRDH) with the technical assistance of ICF Macro and funding from the President's Malaria Initiative (PMI). Malaria control interventions have been implemented in the country recently. The SMIS collected information on interventions such as ownership and use of insecticide treated nets (ITNs) or long lasting impregnated nets (LLINs) as well as intermittent preventive treatment for pregnant women (IPTp). ITN coverage, measured by ownership of at least one mosquito net per household, reached 82% in 2010 [4]. In 2006, Artemisin-based combination therapies (ACTs) were introduced and they were made freely available in 2010. However, indoor residual spraying (IRS) has not been implemented as a routine intervention in the country. During the SMIS only three districts had introduced IRS as a mean of malaria control and therefore no related information was collected in the survey. The number of districts using IRS increased to six in 2010.

A national contemporary map of malaria distribution is an essential tool in order to prioritize control interventions in areas with higher burden and to achieve a better resource allocation and health management. Several maps presenting the distribution of malaria risk in Senegal have been generated over the last few years as part of mapping efforts covering larger areas. A West Africa malaria risk map [5] was obtained using Bayesian geostatistical models on entomological inoculation rate estimates produced by applying the Garki transmission model [6] on historical survey data from the MARA database [7]. An updated malaria risk map for West Africa was estimated using geostatistical models on MARA survey data considering a different effect of environmental factors on malaria depending on the ecological zones [8]. A Senegal malaria risk map was also embedded in a worldwide map based on historical survey data and geostatistical models [9]. All these efforts made use of old and heterogeneous survey data, collected over different seasons, diagnostic tools and overlapping age groups across locations.

Common exposures such as environmental or climatic conditions as well as socio-economic status influence the transmission of malaria similarly in neighboring regions introducing spatial correlation. Geostatistical models including location-specific random effects were employed to model spatial correlation as a function of the distance between sampled locations. The data consisted of a large number of locations with zero prevalence; therefore the commonly used Binomial distribution may underestimate the zero-prevalence probability. Zero-Inflated Binomial (ZIB) models provide a flexible way to address this problem [10]. ZIB models for prevalence data have not been applied before in the context of geostatistical modelling of infectious disease data. To our knowledge, the only application is in the modeling of sparse malaria entomological data [11]. Zero-Inflated Poisson/Negative Binomial models have been formulated for geostatistical count data (i.e. mapping isopod nest burrows [12] and child HIV/TB mortality [13]), however applications are rather limited.

In this paper, we provide spatially explicit burden estimates of malaria in Senegal using the SMIS data and Bayesian geostatistical Zero-Inflated Binomial models based on variable selection methods for spatial data.

## Materials and Methods

### Country Profile

Senegal is located in Western Africa, facing the North Atlantic Ocean between Guinea-Bissau and Mauritania. Its borders run south of the Casamance River and along the Senegal River respectively. The Gambia penetrates more than 320 km into the country, from the Atlantic coast to the centre along the Gambia River which bisects Senegal's territory. Northern Senegal is characterized by a Sahelian ecological zone with semiarid grasslands and acacia savannas. Malaria is unstable hypoendemic and immunity is acquired later in life. A Sudano-Sahelian zone in the centre of the country is dominated by a flat wooded savanna with very few prominent topographical features. Malaria is endemic in this area and immunity is acquired around the age of ten. The southern part of Senegal is occupied by a Sudano-Guinean ecological zone, with annual rainfall exceeding 800 mm. Malaria is hyperendemic and immunity is acquired in the first five years of life. The urban malaria burden is concentrated in the cities of Dakar, Rufisque, Kaolack and Saint-Louis where the anopheles vector density is very low. The high transmission season in Senegal occurs mainly between July and October. However, in the Senegal River delta area, there are two annual peaks of the disease caused by river flooding: one in the rainy and the other in the dry season.

### Ethical statement

Participation in the survey was voluntary and written informed consent was obtained in the local language before questionnaire administration and blood collection for parasitaemia and anemia testing. Individuals were told about the general purpose of the survey, possible risks and benefits of the survey and those presenting malaria parasites and/or anaemia were treated. The survey protocol was submitted to and approved by the Ethical Review Committee at the National Malaria Control Program and the Institutional Review Board (IRB) of Macro International.

### Malaria Data (SMIS 2008–2009)

A nationally representative random sample of 320 clusters and 9600 households was selected through a stratified two-stage sampling procedure. The clusters were the census units (CU) used by the National Agency for Statistics and Demography (ANSD) in the census carried out in 2002 (Recensement Général de la Population et de l'Habitat, RGPH-2002). However, in the three regions of Kaolack, Kolda and Saint-Louis, the health districts served as sampling clusters. At the first sampling stage, 320 clusters were drawn with probability proportional to the number of households in each cluster. The sampling procedure was stratified by the area type (urban/rural) of the clusters: 67.5% of the selected ones were in rural areas and 32.5% in urban areas. At the second sampling stage 30 households were selected randomly from each cluster. Rural areas are slightly overrepresented due to over-sampling in the three regions of Kaolack, Kolda and Saint-Louis. Geographical information is available at cluster level. As part of the final sampling, one every third village was randomly selected and every child between 6 and 59 months of age was tested for parasitaemia. Two tests were performed, RDT and blood smear test [14]. This study is based on the results of microscopic examination since thick blood smear test is considered as the gold standard [15].

### Malaria predictors

**Figure 1:**
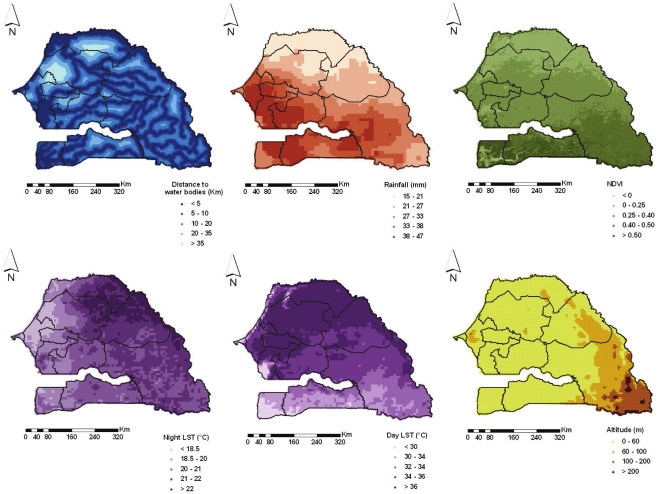
Environmental and climatic factors. Distance to water bodies, Rainfall, NDVI (Normalized Differenced Vegetation Index), Night and Day LST (Land Surface Temperature) and altitude at 4 km^2^ resolution in Senegal. Regional boundaries are overlaid.

Three sets of malaria predictors were considered in the study, namely environmental/climatic proxies, socio-economic factors and malaria intervention measures. The environmental/climatic variables were extracted from remote sensing sources. Decadal rainfall data were downloaded via the Africa Data Dissemination Service (ADDS). Weekly day/night land surface temperature (LST) and biweekly normalized difference vegetation index (NDVI) data were obtained from Moderate Resolution Imaging Spectroradiometer (MODIS). Permanent rivers and lakes were extracted from Health Mapper. The shortest Euclidean distance between the centroid of each pixel and the closest water body was calculated in ArcGIS version 9.1 (ESRI; Redlands, CA, USA). Altitude data were obtained from an interpolated digital elevation model (DEM) developed by the U.S. Geological Survey - Earth Resources Observation and Science (USGS EROS) Data Center. The geographical distributions of the environmental factors are displayed in Figure 1. Data on the rural extents in Senegal are provided by the Global Rural-Urban Mapping Project (GRUMP). According to the UN definition for Senegal, agglomerations with more than 10 000 inhabitants were considered as urban [16]. The above data were available at 1 km^2^ spatial resolution, with the exception of rainfall which has a resolution of 8 km^2^.

Socioeconomic disparities were measured by a wealth index, included in the SMIS data and calculated by a weighted sum of household assets. The weights were estimated through principal components analysis [17]. ITN related information in the SMIS was used to calculate the following ITN coverage indicators ([18] and [19]): i) a binary variable reporting whether the child has a bed net for sleeping; ii) the proportion of children under the age of 5 years reported to have slept under an ITN the night before the survey visit; iii) the total number of nets per household (irrespective of the number of household members); iv) a binary indicator representing the availability of at least one ITN per every two household members and v) at least one ITN per every two children under the age of 5 years in the household. Human population data estimates for the year 2010 were obtained from the Gridded Population of the World version 3 (GPWv3) database at 1 km^2^ spatial resolution. These data were used to convert spatially explicit parasitaemia risk estimates into number of infected children under the age of 5 years. The total number of children under 5 years of age was obtained from the International Data Base of the U.S. Census Bureau, Population Division for the year 2010.

### Bayesian geostatistical modeling

Let 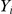 and 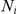 be the number of infected with malaria parasites and the number of screened children under the age of 5 years at location 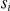 (i.e. cluster centroid) respectively. 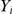 is typically assumed to arise from a Binomial distribution, 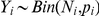 where 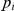 indicates the probability of parasitaemia at 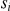. However, in the presence of excessive number of zeros, a Binomial model may be inadequate to estimate the zero-prevalence probability and to identify relevant covariates related to the outcome. To take into account the sparsity of the data, a Zero-Inflated Binomial (ZIB) model 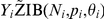 was fitted and compared to the standard Binomial analogue. A ZIB model assumes two sources of zeros: 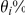 (mixing probabilities) of the zeros are structural, not random and the remaining 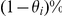 arise with a frequency defined by a Binomial distribution, see equation (1)
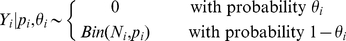
(1)In the above formulation, 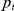 does not have a direct interpretation of parasitaemia risk since it is influenced by the proportion of structural zeros.

The relation between 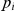 and the vector of 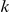 associated predictors 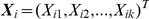 observed at location 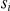 is modeled via the equation 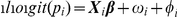, where 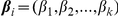 is the regression coefficient vector, 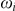 and 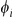 are location-dependent random effects. Spatial dependence is introduced by assuming that the random effects 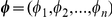 are distributed according to a MVN distribution with mean 0 and covariance matrix 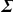 where each element 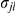 is defined by an exponential parametric function of the distance 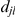 between two locations 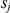 and 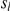, i.e. 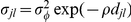. The parameter 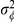 represents the spatial variation and 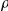 is the parameter controlling the rate of correlation decay with increasing distance. In the case of exponential correlation function, 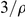 can be used to calculate the distance above which spatial correlation is negligible, known as range. Any remaining non-spatial variation is estimated by the random effects 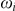, assumed independent and normally distributed with mean 0 and variance 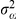.

Bayesian variable selection approaches were employed using the above geostatistical models to choose the best set of predictors. In particular, three variable selection methods, namely Gibbs variable selection (GVS) by Dellaportas et al. [20], Stochastic Search Variable Selection (SSVS) by George and McCulloch [21] and the variable selection sampler of Kuo and Mallick (KM) [22] were compared. The best set of covariates was indicated by the model with the highest posterior probability. Details of the geostatistical variable selection methods are given in the Appendix.

The model includes over 330 parameters. To enable model fit and prediction a Bayesian formulation and MCMC estimation was adopted. To complete model specification, prior distributions were assigned to the parameters. An inverse-gamma prior was assumed for the variance and a gamma distribution for the spatial decay parameter 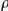. The priors for the regression coefficients were non-informative Gaussian distributions with mean 0 and variance 100. Covariates were standardized in order to acquire better correlation properties and reduce MCMC computational time [23].

Bayesian kriging was employed to predict the parasitaemia risk at unsampled locations and produce a parasitaemia risk map at high spatial resolution [24]. A regular grid of 4 km^2^ resolution covering the whole country was created, resulting in around 60 000 pixels. Predictions were based on a geostatistical model using only environmental/climatic factors since data on malaria interventions or socio-economic status are not available at high resolution scale for the whole country. Therefore, a two stage geostatistical variable selection approach was applied. In the first stage, only climatic predictors were included to identify the best prediction model. In the second stage, geostatistical variable selection was carried out to select among the five ITN coverage indicators defined above. The models were adjusted for age, wealth index and the climatic predictors determined during the first stage.

The predictive model was validated on a test subset of the data. In particular, a randomly selected sample of 269 locations (85% of the data locations) was used as a training set for model fit. The predictive performance of the model was assessed by calculating the proportion of observed prevalence data at the remaining 15% of (test) locations, correctly estimated within Highest Posterior Density Intervals (HPDI) of probability coverage ranging from 50 to 100% [25]. The above validation procedure was also used to compare the ZIB model with its Binomial analogue. The number of malaria infected children under five years of age was estimated at pixel level by multiplying the geostatistical model-based risk estimates with the total number of children under the age of 5 years provided by the International Data Base of the U.S. Census Bureau, Population Division for the year 2010. The previous values were added to calculate the total infected children under the age of 5 years at district level. Subsequently, dividing by the number of children under the age of 5 years living in the district, population-adjusted estimates of parasitaemia risk were obtained.

Fortran 95 (Compaq Visual Fortran Professional 6.6.0) and standard numerical variables (NAG, The Numerical Algorithms Group Ltd.) were used to implement the MCMC code. OpenBUGS [26] was also employed in the model fit.

## Results

A total of 4138 children between 6 and 59 months of age from 320 clusters were tested for parasitaemia with both RDT and blood smear test. The overall observed malaria prevalence was 6.74%. The number of children under the age of 5 years tested with both Rapid Diagnostic Test and blood smear test was 3960. Almost 12.05% of the children under the age of 5 years tested with RDT were found positives. The percentage of children under the age of 5 years that were positives to both tests was 5.44%. Due to the observed discordance between the diagnostic tools, the standard microscopy test was considered in the analysis [15].

**Figure 2:**
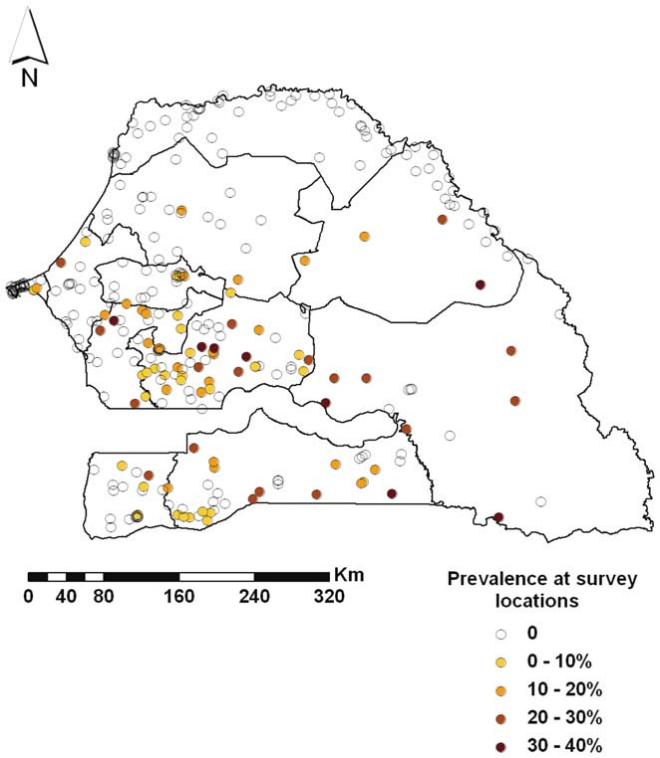
Prevalence at survey locations. Prevalence reported in the 317 locations of the SMIS 2008. Regional boundaries are overlaid.

A large number of survey locations (around 70%) had zero prevalence. No children under the age of 5 years were tested in two clusters of Saint-Louis region and one cluster in Kaolack, thus reducing the actual number of GPS coordinates to 317. Figure 2 shows that the lowest malaria prevalence in the country was recorded in Saint-Louis (0%), followed by the regions of Dakar (1.72%) and Louga (1.43%).

**Figure 3:**
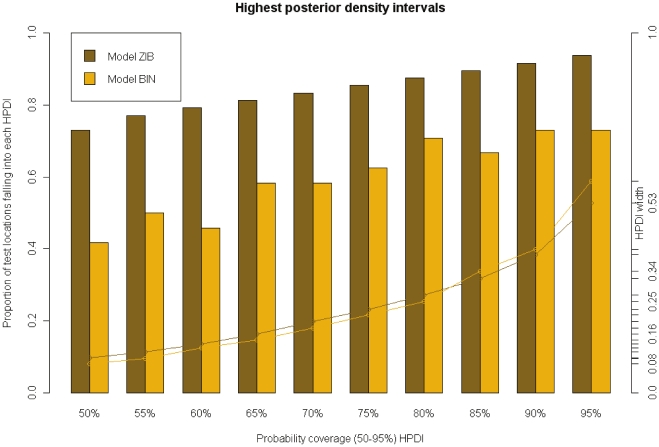
Model comparison and validation. Percentage of test locations with malaria prevalence falling in the highest posterior density intervals (HPDI) predicted from Binomial and Zero-Inflated Binomial models (bars). Lines indicate the corresponding HPDI length.

Posterior model probabilities obtained from MCMC runs of 100 000 iterations using the GVS are presented in Table 1. Similar results were obtained with the other two variable selection methods, SSVS and KM. As shown in the table, the set of covariates that defined the Binomial as well as the ZIB geostatistical models with the highest posterior probabilities consisted of night LST, NDVI and area type (urban/rural). The predictive performance of the selected models is shown in Figure 3. The proportion of test locations falling into the 50–95% HPDIs was constantly higher under the ZIB model. Furthermore, the latter model estimated narrower HPDIs. Based on the above results, the ZIB was adopted to predict the parasitaemia risk at high spatial resolution and to assess the effects of interventions on the infection risk.

**Table 1:** Posterior model probabilities obtained using Gibbs Variable Selection (First stage).

Model	Environmental Variables	Binomial	ZIB
**1.**	Night LST, NDVI	2.46%	2.52%
**2.**	Night LST, NDVI, Area type	72.21%	74.28%
**3.**	Night LST, Rainfall, NDVI, Area type	12.13%	13.23%
**4.**	Others	13.2%	9.97%

In the model with the highest posterior probability (72.21% with Binomial model and 74.28% with ZIB), Night LST, NDVI and Area type were included as covariates. This model was selected and used to predict the malaria risk.

**Figure 4:**
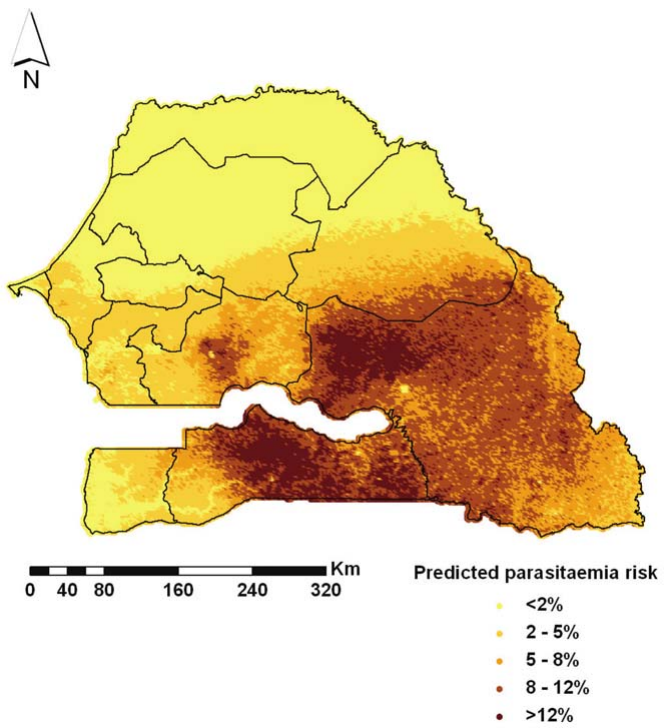
Predicted parasitaemia risk map. Predicted parasitaemia risk in children less than 5 years of age at 4 km^2^ resolution in Senegal. Regional boundaries are overlaid.

Geostatistical ZIB model parameter estimates are given in Table 2. Model I includes only climatic covariates. The posterior estimate of the OR indicates a positive association between NDVI, night LST and parasitaemia, however the corresponding 95% credible intervals include one. Living in urban areas reduces the parasitaemia odds by 81% (95% BCI: 55%–93%). Raw data summaries estimate a parasitaemia prevalence of 1.3% in urban compared to the 8.47% in rural areas. The range parameter suggests that spatial correlation is present up to a distance of 2.4° which is equivalent to 265 km (1° = 111.12 km). The spatial variance (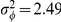) was around 5 times higher than the non-spatial one (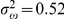) indicating high geographical variation. Model based predictions, obtained through Bayesian kriging over a grid of around 60 000 pixels of 2 km×2 km spatial resolution are depicted in Figure 4. The plotted values correspond to the medians of the pixel-specific posterior predictive distributions. Low values of parasitaemia prevalence are concentrated in the northern Senegal, particularly in the region of Saint-Louis, Louga and Matam. Malaria risk increases in some areas of central Senegal and reaches the highest values in the southern Kolda and eastern Tambacounda where the predicted risk was 10.66% and 9.45%, respectively. Another high-risk area is located in the centre of Kaolak region with an estimated prevalence of 5.6%.

**Table 2:** Association of parasitaemia risk with environmental/climatic factors, socio-economic status and malaria interventions resulting from raw data summaries and geostatistical Zero-Inflated Binomial models.

Variable	Raw Data	Geostatistical model I^a^		Geostatistical model II^b^	
	Prevalence	OR	95% BCI^c^	OR	95% BCI^c^
**Night LST**		1.16	(0.66, 1.86)	0.83	(0.53, 1.26)
**NDVI**		1.48	(0.88, 2.48)	0.91	(0.61, 1.83)
**Area type**					
Rural	8.47%	1		1	
Urban	1.30%	0.19	(0.07,0.45)	0.43	(0.16, 1.06)
**Wealth Index** d					
Most poor	13.75%			1	
Very poor	6.51%			0.77	(0.57, 1.03)
Poor	1.51%			0.22	(0.08, 0.51)
Less poor	0.96%			0.12	(0.05, 0.41)
Least poor	0.65%			0.09	(0.01, 0.26)
**Age**					
0–1	3%			1	
1–2	4.54%			1.20	(0.70, 2.43)
2–3	8.07%			2.93	(1.62, 5.33)
3–4	7.95%			2.96	(1.66,5.74)
4–5	8.11%			2.77	(1.44, 5.21)
**ITNs** e					
<1	6.84%			1	
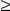 1	1.41%			0.14	(0.03, 0.7)

a Model I includes only environmental/climatic factors.

b Model II includes ITN coverage, children's age and wealth index.

c Bayesian Credible intervals.

d Household wealth index.

e Number of available ITNs per every two household members.

f The range parameter (degrees), defined as 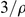 indicates the distance above which the spatial correlation becomes negligible.

**Figure 5:**
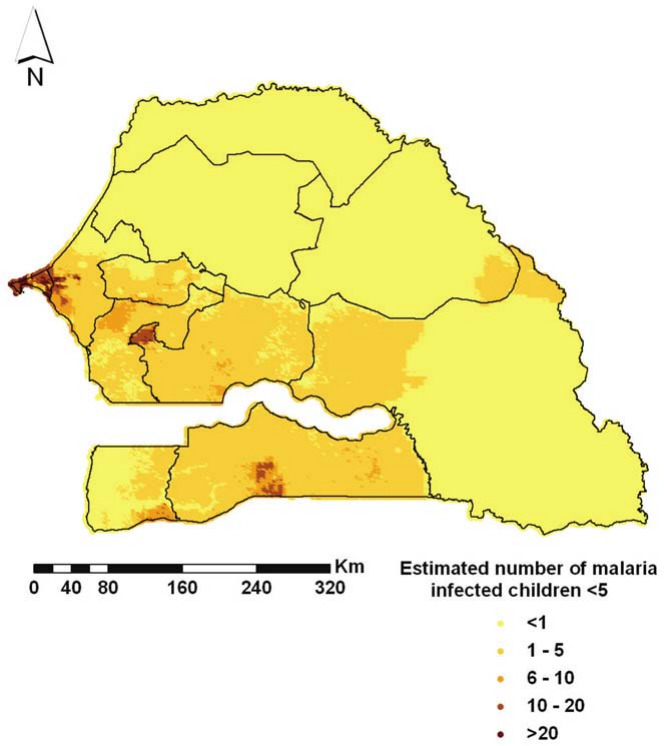
Estimated number of malaria infected children <5 years. The smooth map depicts the estimated number of malaria infected children less than 5 years of age at 4 km^2^ resolution in Senegal. Regional boundaries are overlaid.

The predicted number of malaria infected children under the age of 5 years is displayed in Figure 5 and the estimates of population-adjusted prevalence obtained at the smallest administrative level (*arrondissement*) are summarized in Table 3. Kriging enabled the estimation of parasitaemia prevalence in areas where no survey locations were selected by the sampling procedure. For instance, the population-adjusted prevalence is 0.61% in the *arrondissement* of Barkedji, Louga region and 9.54% in Keniaba, Tambacounda region. The total number of infected children under the age of 5 years in the country below the age of five was estimated to be around 48 thousand. The map of the estimated number of children under the age of 5 years infected with malaria and the predicted parasitaemia prevalence show very different patterns, because of the population density, higher in the urban regions of Dakar and Saint-Louis.

**Table 3:** Estimates of infected children less than 5 years old per *arrondissernent*.

Region	Department	*Arrondissement*	OP^a^	EIC^b^	PEP^c^	Region	Department	*Arrondissement*	OP^a^	EIC^b^	PEP^c^
Dakar	Dakar	Parcelles Assainies	0%	360	0.20%	Louga	Louga	Keur Monar Sarr	2.27%	72	0.31%
Dakar	Guédiawaye	Guédiawaye	0%	112	0.17%	Louga	Louga	Sakal	0%	48	0.13%
Dakar	Pikine	Niayes	0%	356	0.21%	Matam	Matam	Agnam-Civol	0%	376	1.56%
Dakar	Rufisque	Diamnadio	12.5%	272	1.16%	Matam	Matam	Ogo	3.09%	1044	4.07%
Dakar	Rufisque	Rufisque-Bargny	4.35%	72	0.27%	Matam	Ranérou	Ranérou	4.65%	1464	4.29%
Diourbel	Bambey	Baba-Garage	0%	176	0.87%	Matam	Kanel	Sinthiou Bamambé	25%	532	5.08%
Diourbel	Bambey	Lambaye	0%	236	1.17%	Saint-Louis	Dagana	Ross-Béthio	0%	120	0.28%
Diourbel	Diourbel	Ndindy	0%	516	1.41%	Saint-Louis	Podor	Gamadji Sarré	0%	72	0.30%
Diourbel	Diourbel	Ndoulo	0%	384	0.93%	Saint-Louis	Podor	Thillé-Boubacar	0%	80	0.12%
Diourbel	Mbacké	Taif	15.38%	436	1.96%	Saint-Louis	Dagana	Mbane	0%	48	0.28%
Diourbel	Mbacké	Ndame	1.52%	356	0.44%	Tambacounda	Bakel	Moudéry	27.08%	2436	7.49%
Fatick	Fatick	Diakhao	14.29%	420	2.94%	Tambacounda	Bakel	Kéniaba	—	384	9.54%
Fatick	Fatick	Niakhar	8.11%	636	2.75%	Tambacounda	Bakel	Kidira	0%	204	7.13%
Fatick	Fatick	Tattaguine	7.55%	840	2.99%	Tambacounda	Kédougou	Bandafassi	—	244	8.24%
Fatick	Foundiougne	Djilor	7.14%	128	1.94%	Tambacounda	Kédougou	Salémata	—	172	6.89%
Fatick	Foundiougne	Colobane	6.98%	676	2.21%	Tambacounda	Kédougou	Saraya	37.04%	208	6.35%
Fatick	Gossas	Ouadiour	5.17%	540	2.98%	Tambacounda	Tambacounda	Koumpentoum	36.84%	3464	10.49%
Kaolack	Kaffrine	Maka Yop	6.91%	2240	5.53%	Tambacounda	Tambacounda	Koussanar	17.31%	800	4.12%
Kaolack	Kaffrine	Malem Hoddar	11.87%	1740	6.15%	Tambacounda	Tambacounda	Makacoulibantang	0%	1884	10.14%
Kaolack	Kaolack	Sibassor	15.79%	1208	5.07%	Tambacounda	Tambacounda	Missirah	6.67%	424	5.71%
Kaolack	Kaolack	Ndiédieng	5.1%	452	3.01%	Thiès	Mbour	Ndaganiao	—	224	2.47%
Kaolack	Kaolack	Koumbal	4%	884	1.41%	Thiès	Mbour	Sèsséne	5.08%	540	2.62%
Kaolack	Nioro du Rip	Paoscoto	3.77%	1264	3.48%	Thiès	Mbour	Sindia	0%	684	1.84%
						Thiès	Thiès	Keur Moussa	0%	1312	1.31%
Kaolack	Kaffrine	Birkelane	8.2%	656	3.54%	Thiès	Thiès	Thiénaba	—	120	1.12%
Kolda	Kolda	Dabo	39.18%	3212	13.1%	Thiès	Tivaouane	Méouane	1.59%	360	1.09%
Kolda	Kolda	Médina Yoro Foula	27.17%	3240	8.59%	Thiès	Tivaouane	Médina Dakar	0%	156	0.99%
Kolda	Sédhiou	Bounkiling	19.23%	1632	6.84%	Thiès	Tivaouane	Niakhène	0%	288	0.92%
Kolda	Sédhiou	Diendé	3.49%	1280	3.62%	Thiès	Tivaouane	Pambal	27.78%	136	1.71%
Kolda	Sédhiou	Djibabouya	5.56%	460	5.23%	Ziguinchor	Bignona	Sindian	6.25%	408	3.52%
Kolda	Vélingara	Bonconto	5.45%	2644	8.98%	Ziguinchor	Bignona	Tendouck	0%	208	2.19%
Kolda	Vélingara	Kounkané	9.76%	1472	10.47%	Ziguinchor	Bignona	Tenghory	3.33%	196	1.44%
Louga	Kébémer	Ndande	0%	92	0.33%	Ziguinchor	Oussouye	Loudia-Ouoloff	0%	32	1.17%
Louga	Linguère	Barkedji	—	64	0.61%	Ziguinchor	Ziguinchor	Niaguis	—	24	1.37%
Louga	Linguère	Dodji	4.92%	348	1.50%	Ziguinchor	Ziguinchor	Niassia	1.25%	348	0.78%
Louga	Linguère	Yang Yang	0%	68	0.52%	Ziguinchor	Bignona	Diouloulou	6.25%	120	1.72%

a Observed Prevalence.

b Estimated number of infected children under 5 years of age.

c Population-adjusted estimated prevalence.

Data based on the old administrative division (Decret n° 2002-166).

Geostatistical variable selection among the five different ITN coverage indicators (Table 4) showed that having at least one available ITN per every two household members was most related with the parasitaemia risk after adjusting for climatic/environmental factors, age and wealth index. The posterior probability of the model was around 34% indicating that the model was chosen 34% of the times among the 2^5^ = 32 possible models including all combinations of the five coverage indicators. Estimates of the posterior distribution of the parameters are given in Table 2 (Model II). Living in a household with at least one ITN per every two members was found to have a protective effect on parasitaemia, reducing the odds by 86% (95% BCI: 30%–97%). This result was also seen in the raw data summaries as shown by the second column of Table 2. The observed parasitaemia risk in the two categories, i.e. “less than one ITN” and “at least one ITN” per every two members was 6.84% and 1.41% respectively. Posterior estimates of the ORs related to the wealth index show a decreasing trend with the quintiles. The second quintile (very poor) had an OR of 0.77 (95% BCI: 0.57–1.03) whereas the last one (least poor) was 0.09, (95% BCI: 0.01–0.26). A similar pattern was presented in the prevalence calculated from the raw data. The highest (13.75%) and lowest (0.65%) infection risk were observed in the most and least poor group, respectively. Infection odds appear to be increasing with age. For instance, the OR is 1.2 (95% BCI: 0.70–2.43) in children 1–2 years old and 2.77, (95% BCI: 1.44–5.21) in children 4–5 years old. Observed parasitaemia prevalence was the lowest in infants (3%) reaching 8.11% in children 4–5 years old.

**Table 4:** Posterior model probabilities obtained using Gibbs Variable Selection (Second stage).

Model	ITN coverage indicators	Posterior Probabilities
**1.**	None	25.20%
**2.**	Ownership of 1 ITN per 2 household members	34.00%
**3.**	Child has ITN for sleeping, ownership of 1 ITN per 2 household members, n. of ITNs per household	7.80%
**4.**	Others	33.0%

The model with the highest posterior probability (34%) includes “Ownership of 1 ITN per 2 household members” as the selected ITN coverage indicator.

## Discussion

This study estimated the number of infected children under the age of 5 years at different geographical scales in Senegal and produced the first parasitaemia risk map in the country using contemporary data collected under the nationally representative malaria survey of 2008/2009. Geostatistical Zero-Inflated Binomial models were developed and Bayesian variable selection methods for spatially correlated data were employed to build a predictive model and assess the effectiveness of the ITN intervention adjusting for climatic and socio-economic confounders.

A large number of zeros was observed when modeling the number of infected children under the age of 5 years, probably due to the fact that the survey was carried out at the beginning of the dry season, when transmission starts to decrease. To address the issue of sparsity a ZIB model was derived. Model validation revealed that the ZIB model had higher predictive ability than the Binomial analogue suggesting that, when a large number of zeros occurs in the data, a ZIB model should be considered. Since malaria research is focused on elimination and eradication of the disease, it is expected that forthcoming surveys will include a large number of locations with zero prevalence and the ZIB models would provide a suitable alternative to the standard Binomial ones for geostatistical modeling.

Geostatistical variable selection is an important topic in malaria mapping. The predictive ability of a model depends on the covariates included in the multivariate regression setting. Modeling approaches in malaria mapping treat selection of predictors separately than the geostatistical model fit. Variable selection is often based on regression models that ignore spatial correlation, leading to wrong estimates of covariates effects and their significance. Geostatistical variable selection not only identifies the best set of predictors but builds parsimonious models with the best predictive ability [27]. In addition, it can be used to avoid overfitting due to the inclusion of unnecessary predictors or random effects. In this work, we have employed three Bayesian variable selection methods within a geostatistical model formulation. The climatic model with the highest posterior probability selected by the three methods included the following combination of covariates: night LST, NDVI and area type. Altitude in Senegal presents very little variation throughout the country therefore it was not considered as a potential predictor of malaria transmission in the variable selection procedure.

As mentioned above, maps showing the distribution of malaria risk in Senegal can be found in [5], [8] and [9] as part of efforts in mapping malaria risk at regional and continental level using historical data. Nevertheless, compilations of historical data obtained from surveys, heterogeneous in the age groups involved and the seasons considered, require methods for standardizing risk estimates into a common scale for mapping purposes. Different statistical methods have been employed; the work by Gemperli and colleagues [5], for instance, made use of the Garki transmission model to take into account the heterogeneity in the surveys. The model developed by Pull and Grab [28] was instead employed by the MAP project [9], standardizing age-groups to produce a world map of Plasmodium falciparum malaria endemicity. The parasitaemia risk map presented in this paper, has been estimated from a contemporary survey and shows similar patterns to the one obtained from previous efforts [5], especially in the Southern and Eastern part of Senegal, at the border with Mali where the risk is higher. However, Gemperli et al. [5] predicted a lower risk in the Central part of the country and higher in the urban areas of Dakar and Saint-Louis, as well as throughout the Sahelian region. In terms of absolute values, those results are uniformly higher than the current ones, due to the fact that the SMIS was carried out at the beginning of the low transmission season. The predicted pattern of malaria produced by the more recent work by Gosoniu et al. [8] is more consistent with the map we generated, however the absolute values are still far from our estimates. The map of Senegal from the MAP project [9] does not show any relevant variations or geographical differences in the intensity of malaria risk throughout the country. For logistic reasons the survey took place at the start of the dry season, thus projections from our model are likely to underestimate the burden during the highest transmission season.

Furthermore, the differences between observed and population adjusted risk estimates are mainly due to low prevalence observed in highly populated areas. The urban area of Dakar, for example, is the most populated one, and the majority of surveys were carried out in that area although the parasitaemia risk is very low.

Geostatistical variable selection enabled the assessment of the effect on parastaemia risk of different ITN coverage indicators after taking into account climatic factors and socio-economic disparities. Recent work by [18] and [19] proposed a number of ITN coverage measures related to the ownership and use of nets at individual or household level. Five indicators have been assessed in the study and only one suggested a reduction in malaria risk with increasing coverage. This may explain the lack of relation between ITN coverage and malaria risk in similar analyses of MIS data. The Senegal data revealed that the presence of at least one ITN per every two household members reduced the odds of parasitaemia by 86%. In a recent analysis in Tanzania [29], ownership of at least one ITN was the only indicator assessed, showing no protective effect. On the other hand, the analysis of Zambia MIS 2005 [30] measured ITN coverage by the ownership of at least one bednet per household and found a preventive effect on malaria risk. Gosoniu et al. [31] reported a reduction in risk for areas having at least 0.2 ITNs per person, a measure similar to the one presented in this paper. Different indicators of ITN coverage were considered in a spatial analysis of the Liberia MIS data [27], however none of them was associated with a reduction in the infection risk.

The model does not include some known risk factors for malaria such as maternal education, proximity to health services as this information was not readily available from the MIS data. It is however interesting to collect this data and include them in future MIS analyses aiming to assess ITN effects on parasitaemia.

This study found that the malaria risk in children less than five years old increases with age. Infants had the lowest risk. The risk rises especially after the age of two and levels off in older children. Similar results were observed in other low endemic settings.

All the results presented in the paper are based on the estimation of parasitaemia prevalence using the blood smear test. Malaria prevalence estimated using the RTDs was almost twice as high as the one based on the microscopy results. This confirms earlier findings suggesting that RDTs might present a large number of false positives when used in field conditions probably due to high temperatures during storage and transport as well as poor training on RDTs use.

In the model formulation, a linear relation between the parasitaemia odds and the environmental covariates was assumed. Geostatistical variable selection could be used to determine the best functional form that describes the above relation. Furthermore, a stationary geostatistical model was fitted assuming that spatial correlation depends only on the distance between locations irrespective of the locations themselves. This assumption may not be true when there are unobserved factors, such as health system performance, that vary across the country. The relation between climatic predictors and malaria may differ as well among the ecological zones.

Future control interventions can be planned and implemented by decision-makers according to the priority of the areas. A better resource allocation and health management can be achieved by monitoring the impact of prevention and control activities. The produced map and estimates generated in this study can be considered as baseline for comparisons with future national surveys to evaluate the effectiveness and progress of on-going intervention programmes as well as the changes of the parasitaemia risk over space and time.

## Supporting Information

Appendix S1 **Bayesian Geostatistical variable selection methods.**
(DOC)
