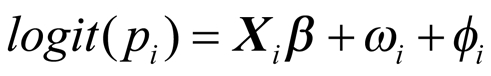# Correction: Estimating the Burden of Malaria in Senegal: Bayesian Zero-Inflated Binomial Geostatistical Modeling of the MIS 2008 Data

**DOI:** 10.1371/annotation/e7549f68-308c-45d5-a14d-8b642a930495

**Published:** 2012-04-17

**Authors:** Federica Giardina, Laura Gosoniu, Lassana Konate, Mame Birame Diouf, Robert Perry, Oumar Gaye, Ousmane Faye, Penelope Vounatsou

There was an error in formatting the equation in the first sentence of the second paragraph of the "Bayesian geostatistical modeling" section of Materials and Methods. The correct equation can be viewed here: