# Supplementary material for: Estimating the Burden of Malaria in Senegal: Bayesian Zero-Inflated Binomial Geostatistical Modeling of the MIS 2008 Data
Source: PLoS One. 2012 Mar 5;7(3):e32625. doi: 10.1371/journal.pone.0032625 (PMC3293829; doi:10.1371/journal.pone.0032625)
Supplement: Appendix S1 — Bayesian Geostatistical variable selection methods. (DOC) [file pone.0032625.s001.doc]

**Appendix S1**

Estimating the burden of malaria in Senegal:

Bayesian Zero-Inflated Binomial geostatistical

modeling of the MIS 2008 data

Federica Giardina, Laura Gosoniu, Lassana Konate, Mame Birame Diouf,

Robert Perry, Oumar Gaye, Ousmane Faye and Penelope Vounatsou

**Bayesian Geostatistical variable selection methods**

Given a vector of $k$ potential regressors $X=(X_{1},X_{2},...,X_{k})^{T}$, we aim at selecting the "best" subset $X^{*}=(X_{1}^{*},X_{2}^{*},...,X_{q}^{*})^{T}\text{ }q\leq k$ to model the standard Binomial component $p_{i}$ of the Zero-Inflated model. To this purpose, the geostatistical model was modified to let the MCMC scheme choose among the $2^{k}$ models: an auxiliary indicator variable $g_{j}$ was introduced, where $g_{j}=1$ indicates presence and $g_{j}=0$ indicates absence of covariate $j$ in the model. The prior that was used for the indicator $g_{j}$ is $g_{j}\sim Bernoulli(1/2)$ , i.e. the probability of inclusion in the model for each variable is 0.5.

The three different formulations of Bayesian Variable selection strategies in Geostatistical models implemented in the work are described and compared below.

*Gibbs Variable Selection*

The method relies on a linear predictor defined by the equation

where $g_{j}$ is the indicator defined in the previous paragraph. A mixture of independent normal distributions $\beta_{j}\sim N(0,\sigma_{j}^{2})+(1-g_{j})N(\bar{\mu}_{j},\tau_{j}^{2}),\text{ }j=1,2,...,k$ was used as a prior for the coefficients where $\sigma_{j}^{2}$ is the prior variance when the $j-th$ term is included in the model and $\bar{\mu}_{j}$ and $\tau_{j}^{2}$ are the mean and the variance respectively used when the $j-th$ term is not included in the model (pseudoprior).

*Kuo & Mallick*

The most straightforward method for variable selection has been proposed by Kuo and Mallick [15]. The method assumes that the indicators and the covariates effects are a priori independent, i.e. $f(\beta_{j},g_{j})=f(\beta_{j})f(g_{j}),\text{ }j=1,2,...,k$. It is easy to implement and requires only the specification of the prior distribution for the regression coefficients, usually assumed to be non informative Gaussian . The relation between the predictors and the outcome is given by equation (2), as for the Gibbs Variable Selection method.

*Stochastic Search Variable Selection*

The SSVS method is slightly different since the parameter vector retains its full dimension $k$ of all potential covariates under all models. It assumes a prior distribution for the coefficients composed by a mixture of Normal distribution $\beta_{j}\sim N(0,\sigma_{j}^{2})+(1-g_{j})N(0,l_{j}^{-2}\tau_{j}^{2}),\text{ }j=1,2,...,k$ where $l_{j}$ is specified in order to ensure that the coefficient $\beta_{j}$ is close to 0 when $g_{j}=0$, i.e. the $j-th$ variable is not included in the model.

In particular, the linear predictor is given by equation .

For a comprehensive review of these methods, see [32] while a simplified version for practitioners could be found in [27].
